# Effects of thymic stromal lymphopoietin on cord blood progenitor cell differentiation and hemopoietic cytokine receptors expression

**DOI:** 10.1186/1710-1492-7-S2-A24

**Published:** 2011-11-14

**Authors:** Claudia CK Hui, Ilan Asher, Delia Heroux, Zoulfia Allakhverdi, Guy Delespesse, Judah A Denburg

**Affiliations:** 1 McMaster University, Hamilton, Ontario, Canada; 2 CRCHUM, Notre-Dame Hospital, Montreal, Quebec, Canada

## Background

TSLP has been described as a “master T_H_2 cytokine” with pleiotropic effects. Recent research shows that TSLP can directly activate mast cells and CB CD34^+^ hemopoietic progenitor cells (HPC), with release of T_H_2 cytokines/chemokines in a TSLP-dependent manner [1,2]. Additionally, TSLP may modulate the function of CD34^+^ cells via changes in hemopoietic cytokine receptors (HCR), which are altered in CB CD34^+^ cells of atopic at-risk infants [3]. In this study, we aimed to characterize HCR expression on CD34^+^ cells and to examine CD34^+^ cell differentiation following TSLP stimulation.

## Methods

Purified CD34^+^ HPC isolated from CB were stimulated with rTSLP (0.1, 0.5, and 1 ng/ml), with or without IL-33 then stained for surface expression of HCR, or used for methylcellulose colony assays. Flow cytometric staining of CD34^+^ cells were performed using monoclonal antibodies to CD34, CD45, polyclonal rabbit antibodies to IL-3Rα, IL-5Rα, and GM-CSFRα, and compared to isotype controls. CD34^+^ cells were cultured in semisolid methylcellulose culture in the presence of optimal doses of recombinant cytokines, IL-3, IL-5, and GM-CSF. Following 14 days of culture, mean numbers of Eo/B CFU were enumerated.

## Results

Overnight stimulation with TSLP (1 ng/mL) significantly enhanced mean expression of IL-5Rα on CB CD34^+^ cells (n=8, p=0.04) and significantly increased Eo/B CFU production to IL-5 and GM-CSF (n=6, p<0.05). Additionally, TSLP sufficiently induced the differentiation of CD34^+^ progenitors into eosinophils.

## Conclusion

Our findings suggest key roles for TSLP in influencing CB HPC eosinophilic lineage commitment eventuating in allergic inflammation and disease in early life.
